# Effects of endocrine disrupting chemicals and plasma metabolome on female reproductive diseases: A multidimensional Mendelian randomization study

**DOI:** 10.1097/MD.0000000000047248

**Published:** 2026-01-16

**Authors:** Boyu Yin, Chunyu Li, Yi Pei, Huafang Wei, Lin Liang, Chuchao Zhu, Chengwen Song

**Affiliations:** a Department of Obstetrics and Gynecology, The PLA General Hospital of Central Command Theater, Wuhan, Hubei Province, China; b Department of Medical Records, Tongji Hospital, Tongji Medical College, Huazhong University of Science and Technology, Wuhan, Hubei Province, China.

**Keywords:** endocrine disrupting chemical, female reproductive disease, Mendelian randomization study, plasma metabolome

## Abstract

Endocrine disrupting chemicals (EDCs) have been associated with disorders affecting female reproductive health, although the precise causal connections and underlying pathways between these substances and such disorders remain incompletely elucidated. Our research implemented multidimensional Mendelian randomization techniques to assess potential causal associations between EDCs and female reproductive diseases (FRDs), while simultaneously evaluating the intermediary functions of metabolites in plasma. Harnessing summary data, we implemented inverse variance weighting method (IVW) as our principal analytical framework to explore causal relationships between 15 EDCs and 11 prevalent FRDs. Additionally, we deployed 4 supplementary methodologies, encompassing MR-Egger regression, weighted median, simple model, and weighted model, to conduct extended investigations. To ensure robustness, we used Cochran’s *Q* test, MR-Egger regression, Mendelian randomization pleiotropy residual sum and outlier, and the leave-one-out method to conduct sensitivity analyses. Moreover, comparable analytical strategies were employed to explore the intermediary contributions of plasma metabolites. Four EDCs showed causal associations with FRDs. Specifically, mono-(2-ethyl-5-carboxypentyl) phthalate was found to increase gestational hypertension susceptibility (OR = 1.541, 95% confidence interval [CI]: 1.063–2.232, *P* = .022), whereas mono-methyl phthalate demonstrated heightened associations with endometriosis occurrence (OR = 1.058, 95% CI: 1.013–1.104, *P* = .011) and pregnancy-induced hypertensive conditions (OR = 1.113, 95% CI: 1.001–1.238, *P* = .047). Furthermore, mediation analysis identified 13 plasma metabolites that mediated EDCs-FRDs associations, including sulfated steroids (e.g., 5α-androstan-3α,17β-diol disulfate) and lipid derivatives. The other 4 auxiliary methods yielded consistent directional estimates. EDCs indicate evidence consistent with causal effects on FRDs risk. Plasma metabolites may represent essential intermediary regulators in the causal pathway between EDCs and FRDs. These findings advocated reducing EDCs exposure and targeting metabolic pathways for FRDs prevention.

**Key Points**

1. Endocrine disrupting chemicals indicate evidence consistent with causal effects on female reproductive disease risk.
2. Plasma metabolites may represent essential intermediary regulators in the causal pathway between endocrine disrupting chemicals and female reproductive diseases.
3. The findings provide robust genetic evidence for preventing female reproductive diseases by reducing exposure to endocrine disrupting chemicals.

## 1. Introduction

Endocrine-disrupting chemicals (EDCs) represent a unique environmental pollutant prevalent in the contemporary environment,^[1]^ which can be detected in popular consumer products such as plastics, pesticides, personal care products, and food packaging materials.^[2,3]^ By structurally resembling endogenous hormones, EDCs can agonize, antagonize, or modulate the biosynthesis and catabolism of physiological hormones, thereby inducing aberrant hormonal signaling that disrupts homeostatic endocrine regulation through either amplification or suppression of normal physiological processes.^[4]^

Recent research has underscored numerous links between EDCs and a variety of negative health effects, such as metabolic issues, disorders related to neurodevelopment, dysfunctions of the immune system, and cancers influenced by hormones.^[5,6]^ A significant concern is how susceptible the female reproductive system is to disruptions induced by EDCs, given that it depends on accurate hormonal control and exhibits heightened sensitivity to environmental toxins.^[7]^

Numerous epidemiological and mechanistic investigations have established connections between EDCs and various female reproductive diseases (FRDs), such as endometriosis (EMS), leiomyomas, polycystic ovary syndrome (PCOS), early ovarian failure, irregularities in menstrual cycles, gestational hypertension, and subfertility, among others.^[8]^ For instance, a study in Alberta identified a significant association between mono-(2-ethyl-5-carboxypentyl) phthalate (MECPP) exposure and pregnancy-induced hypertension (PIH).^[9]^ Similarly, a Chinese case-control study reported a notable link between urinary mono-methyl phthalate (MMP) levels and EMS.^[10]^ An observational analysis conducted in France suggested that maternal exposure to certain phthalates – particularly mono-ethyl phthalate (MEP) – significantly contributes to PIH risk.^[11]^ Moreover, developmental or adult exposure to dioxins has been shown to epigenetically reprogram endometrial tissue, potentiating EMS pathogenesis through inflammatory pathway activation and estrogen receptor hyperresponsiveness.^[12]^ Similarly, phthalates compromise follicular maturation and steroidogenic enzyme activity in granulosa cells, mechanistically contributing to ovarian dysfunction and infertility.^[13]^ These exposures may further elicit systemic endocrine disruptions, manifesting as spontaneous pregnancy loss, preterm labor, and fetal growth restriction, likely mediated via placental vascular dysfunction and oxidative stress amplification.^[14]^ Moreover, parabens exhibit dual disruption of ovarian hormone synthesis and hypothalamic-pituitary-ovarian axis signaling, exacerbating their detrimental effects on reproductive and endocrine homeostasis.^[15]^

EDCs affect female reproductive health through complex mechanisms that may involve multiple pathways, including hormonal interference, epigenetic modification, inflammation, and so on.^[16,17]^ Studies have shown that phthalate exposure interferes with lipid metabolism, leading to elevated arachidonic acid levels, a disorder that may hinder ovarian steroid synthesis and affect endometrial receptivity. Perfluoroalkyl sulfonates also interfere with the β-oxidation of fatty acids and mitochondrial action in granulosa cells, which may also contribute to PCOS.^[18]^ Since perfluoroalkyl sulfonates may interfere with β-oxidation of fatty acids and mitochondrial function in the granulosa cells, they could be a cause for PCOS.^[19,20]^ These results indicated that metabolites in the bloodstream may act as indicators and mechanisms of action, connecting EDCs to FRDs.

Although some improvements have been reached concerning the impact of EDCs on FRDs, these studies have their own deficiencies. Most of them were observational studies and were often disturbed by confounding factors and reverse causal relationships. It was difficult to draw the cause–effect relation between EDCs and FRDs. In addition, the role of blood metabolites in the relationship between EDCs and FRDs remains unclear. To fill these gaps, a robust approach was needed to explore the direct and indirect effects of EDCs on FRDs. One of the effective methods was Mendelian randomization (MR), which showed great promise.

Later in 1986, Katan applied this technique, MR, to probe causal linkages assumed between exposure factors and outcomes using genetic variations as instrumental variables (IVs).^[21]^ By randomizing genetic variants, MR inherits the natural distribution of genetic variants during meiosis, which shares the same randomization structure as a randomized control trial.^[22]^ Additionally, this methodology effectively mitigates problems such as confounding bias and reverse causality that plague many findings in observational research.^[23]^

Therefore, the objective of this study was to investigate the link between EDCs and FRDs, while identifying the role of blood metabolites as possible mediators in these relationships. Through this research, we aimed to clarify the mechanisms behind reproductive harm caused by EDCs and contribute to the understanding and safeguarding of female reproductive well-being.

## 2. Materials and methods

### 2.1. Study design

A multidimensional MR approach was used to explore the relationships between 15 EDCs and 1400 metabolites present in plasma in relation to 11 FRDs. The exposure was firstly established using each of the 15 EDCs, and instrumental variables were chosen based on the principles of correlation, independence, and exclusion criteria.^[24]^ Following this, the MR was applied to measure the associations between EDCs and FRDs. In the final phase, the MR technique was employed to examine how the plasma metabolome mediates the link between EDCs and FRD using comparable analytical strategies.

### 2.2. Data sources

#### 2.2.1. Endocrine disrupting chemicals

Summary data on the concentrations of 15 EDCs in 24-hour urinary excretion were derived from a Genome-Wide Association Study (GWAS) that was published in 2024.^[25]^ This study included 1085 unrelated adults (aged 18–81 years) from the Lifeline cohort.^[25]^ The participants in this study had a broad age spectrum and exhibited a variety of lifestyles, which reduces the bias that may arise when analyzing only high-risk groups, greatly improving the quality and representativeness of the data. These EDCs, including MECPP, MEP, MMP, bisphenol A (BPA), etc, were selected on the based of their availability in the dataset.^[24]^

#### 2.2.2. Female reproductive diseases

Summary data on 11 FRDs were derived from FinnGen Release 11 (R11),^[26]^ accessible at https://www.finngen.fi/en.^[27]^ FinnGen R11 is one of the latest comprehensive genomic datasets derived from over 4,00,000 Finnish participants, which combines genetic data with detailed health registry information. FinnGen provides publicly accessible GWAS summary statistics, enabling researchers to explore genetic associations across a wide range of phenotypes.^[28,29]^ The spectrum of female reproductive system disorders encompassed premature decline in ovarian function, uterine endometrial tissue displacement, ovarian cyst syndrome, compromised fertility, and gestational complications (including PIH, gestational diabetes mellitus, spontaneous abortion, placental abruption, placenta previa [PP], ectopic pregnancy and hydatid mole [HM]).

#### 2.2.3. Plasma metabolome

Information regarding plasma-based metabolic compounds was extracted from a GWAS published in 2023, which documented 1091 blood metabolites along with 309 metabolite ratios.^[30]^ This comprehensive genomic analysis incorporated 8299 participants of European ancestry, selected through randomized methodology from a broader cohort exceeding 50,000 individuals between 45 and 85 years of age participating in the Canadian Extended Temporal Investigation on Aging.^[24]^ To conduct the GWAS analysis, adjustments were made for features such as age, gender, the time since the last food or drink consumed and so on. It also excluded folks with close family ties to increase the quality and decrease bias and the reliability of the study results.

### 2.3. Data extraction

For genetic variants to function effectively as instrumental variables (IVs), 3 fundamental criteria must be satisfied: the single nucleotide polymorphisms (SNPs) must demonstrate robust correlations with the exposure variable; these SNPs should remain unaffected by confounding elements that can impact both the exposure and the outcome; the influence of these SNPs on the outcome should be exclusively mediated through the exposure mechanism without independent pathways, specifically avoiding direct pleiotropic effects.^[21,31,32]^ Consequently, the selection of IVs in this study adhered to the following procedural framework. First, to expand the pool of qualified IVs, SNPs that showed significant associations with the exposure were extracted from GWAS utilizing a significance threshold of $P<5\times 10^{-6}$^[33]^. To guarantee independence among the chosen IVs, a rigorous clump window ($r^{2}\;=\;0.001$ and  kb =10,000) was implemented to eliminate SNPs experiencing linkage disequilibrium.^[24]^ To minimize weak instrument bias effects, genetic variants with *F* statistics below 10 were excluded from consideration.^[24,33]^ Subsequently, an evaluation was conducted to identify and remove SNPs linked to potential confounding variables through the Ensemble platform, taking into account factors such as body mass index, waistline measurement, hip dimension, body weight, menstruation onset age, menopausal transition timing, tobacco usage, and educational attainment.^[24]^ The final screening process employed a threshold of $P<5\times 10^{-6}$ to eliminate SNPs strongly connected to outcomes, thereby ensuring absence of direct relationships between the genetic variants and the observed results.

### 2.4. Statistical analysis

We first employed MR to investigate associations between 15 EDCs and 11 FRDs. Beta coefficients (β) and standard errors for SNP-exposure and SNP-outcomes were used to estimate  β and standard error values along with 95% confidence interval (CI). The fundamental analytical approach implemented was inverse variance weighting method (IVW), which combines the estimates of multiple studies by assigning a weight inversely proportional to the variance estimated by each study, resulting in a more accurate overall estimate. Additionally, 4 complementary MR verification techniques, including MR-Egger regression, simple mode, weighted median and weighted mode, were applied to explore the stability of the results.^[34]^

In the sensitivity analysis, we scrutinized heterogeneity through the Cochran’s *Q* test, with significance thresholds of *P* < .05 designated to signify heterogeneity.^[35]^ The regression intercept in the MR-Egger test and the residual sum of squares observed with the Mendelian randomization pleiotropy residual sum and outlier method were used to test for pleiotropy,^[24]^ which, if showing a statistically significant result, would indicate the presence of pleiotropy in the study. Concurrently, the leave-one-out method was employed to assess the heterogeneity of the effects of SNPs. This method determined the impact of each individual SNP on the outcome by successively eliminating 1 SNP at a time and then computing the causal effect based on the remaining SNPs.

Regarding EDCs that demonstrated statistically significant correlations with reproductive health disorders, we conducted additional investigation into the intermediary functions of plasma metabolites on the connection between EDCs and FRDs. First, we evaluated the relationships between EDCs that were statistically associated with FRDs and 1400 plasma metabolites. We then evaluated the associations between plasma metabolites and FRDs, where plasma metabolites have been shown to have significant associations with EDCs. We used the IVW method to estimate 3 key coefficients: the total effect of EDCs on a particular FRD ($\beta_{all}$); the contribution of EDCs on the plasma metabolome ($\beta_{1}$); and the effect of the plasma metabolome on a certain FRD ($\beta_{2}$).^[24]^ To evaluate the mediating role of a given plasma metabolome, we computed the product of $\;\;\;\beta_{1}$ and $\;\;\;\beta_{2}$. This product allowed us to determine the extent to which the plasma metabolome mediated the relationship. To ascertain the direct effect of EDCs on a specific FRDs, we subtracted the product of $\;\;\;\beta_{1}$ and $\;\;\;\beta_{2}$ from $\;\;\;\beta_{all}$. The resulting value represented the direct effect of EDCs on that particular FRD, independent of the mediating influence of the plasma metabolome.

This study was performed via R software (version 4.0.5) with packages including TwoSampleMR (version 0.6.6), MRPRESSO (version 1.0), data.frame (version 1.16.0), dplyr (version 1.1.4), tidyr (version 1.3.1), and ggplot2 (version 3.5.1). *P* < .05 indicated statistical significance.

## 3. Results

**Figure 1.**
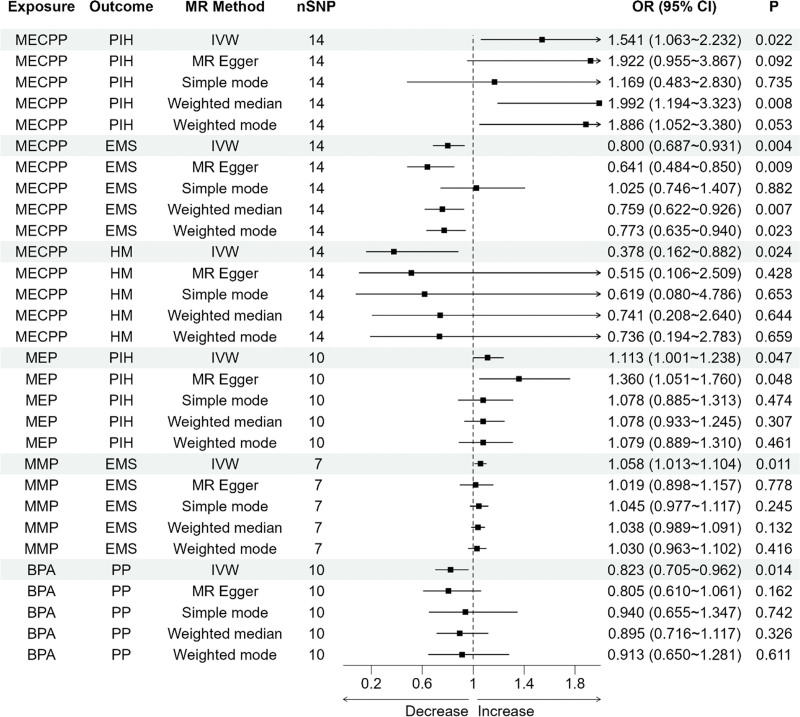
MR estimates of the effects of EDCs exposure on FRDs. This visualization exclusively presents findings where statistically significant associations were identified through application of the IVW methodology. OR (95% CI) and *P* metrics were calculated for the corresponding MR analyses. BPA = bisphenol A, CI = confidence interval, EDC = endocrine disrupting chemical, EMS = endometriosis, FRD = female reproductive disease, HM = hydatid mole, IVW = inverse variance weighted, MECPP = mono-(2-ethyl-5-carboxypentyl) phthalate, MEP = mono-ethyl phthalate, MMP = mono-methyl phthalate, MR = Mendelian randomization, nSNP = number of single nucleotide polymorphism, OR = odds ratio, PIH = pregnancy-induced hypertension syndrome, PP = placenta previa.

Based on the IVW method, we found that there were 4 kinds of EDCs that were causally correlated with FRDs, as shown in Figure 1. Specifically, MECPP increased the risk of PIH (OR = 1.541, 95% CI: 1.063–2.232, *P* = .022) and decreased the risk of EMS (OR = 0.800, 95% CI: 0.687–0.931, *P* = .004) and HM (OR = 0.378, 95% CI: 0.162–0.882, *P* = .024). MEP was found to elevate the risk of PIH (OR = 1.113, 95% CI: 1.001–1.238, *P* = .047). Meanwhile, MMP increased the risk of EMS (OR = 1.058, 95% CI: 1.013–1.104, *P* = .011). BPA, on the other hand, reduced the risk of PP (OR = 0.823, 95% CI: 0.705–0.962, *P* = .014). The findings from additional 4 MR approaches showed directional consistency with the IVW method.

**Figure 2.**
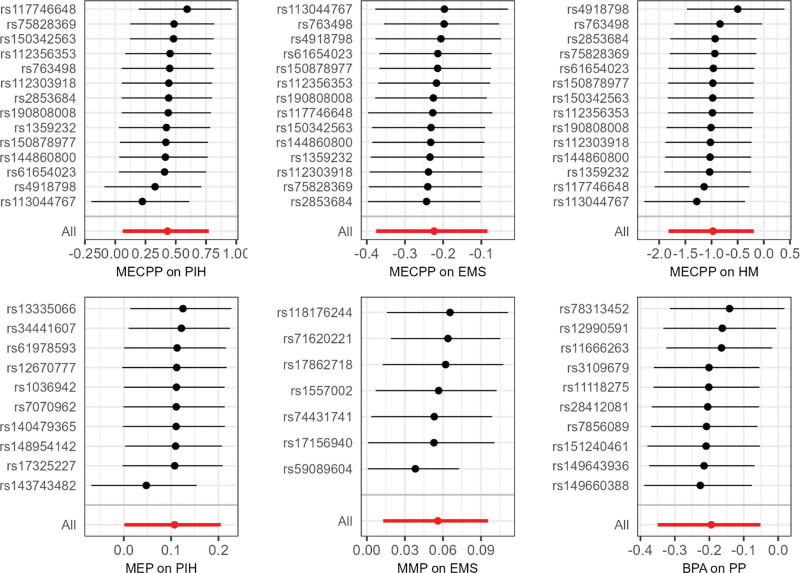
Results of leave-one-out test of the effects of EDCs exposure on FRDs. This figure only showed results where a statistically significant relationship was found based on the IVW method. BPA = bisphenol A, EDC = endocrine disrupting chemical, EMS = endometriosis, FRD = female reproductive disease, HM = hydatid mole, IVW = inverse variance weighted, MECPP = mono-(2-ethyl-5-carboxypentyl) phthalate, MEP = mono-ethyl phthalate, MMP = mono-methyl phthalate, PIH = pregnancy-induced hypertension syndrome, PP = placenta previa.

Table 1 provides summary results of the heterogeneity and pleiotropy tests. The Cochran’s *Q* test indicated no evidence of heterogeneity. Moreover, the MR-Egger and PRESSO tests suggested an absence of pleiotropy. Figure 2 illustrates the results of leave-one-out test. Notably, no SNPs were found to significantly influence the overall results. Collectively, the findings from the heterogeneity, pleiotropy, and leave-one-out analyses reinforce the robustness of the results.

**Table 1:** Heterogeneity and pleiotropy tests of the MR estimates.

Exposure	Outcome	Heterogeneity		Egger pleiotropy		PRESSO global pleiotropy		
		*Q*	*P*	Intercept	*P*	RSSobs	*P*	Outliers
MECPP	PIH	14.135	.364	-0.018	.475	18.833	.325	None
MECPP	EMS	11.414	.576	0.018	.093	12.391	.663	None
MECPP	HM	9.322	.748	-0.025	.658	12.927	.654	None
MEP	PIH	6.797	.658	-0.047	.134	9.429	.621	None
MMP	EMS	9.068	.170	0.015	.567	12.125	.252	None
BPA	PP	8.010	.533	0.006	.847	10.018	.528	None

This table only showed results where a statistically significant relationship was found based on the IVW method. *Q* represented *Q* statistic of heterogeneity test. RSSobs represented residual sum of squares observed.

BPA = bisphenol A, EMS = endometriosis, HM = hydatid mole, MECPP = mono-(2-ethyl-5-carboxypentyl) phthalate, MEP = mono-ethyl phthalate, MMP = mono-methyl phthalate, MR = Mendelian randomization, PIH = pregnancy-induced hypertension syndrome, PP = placenta previa, RSSobs = residual sum of squares observed.

Additionally, the results of reverse causality analysis between the aforementioned associations were shown in Figures S1, S2 and Table S1, Supplemental Digital Content, https://links.lww.com/MD/R173; https://links.lww.com/MD/R174. These findings indicated that there was no reverse causality in any of the above positive associations.

**Figure 3.**
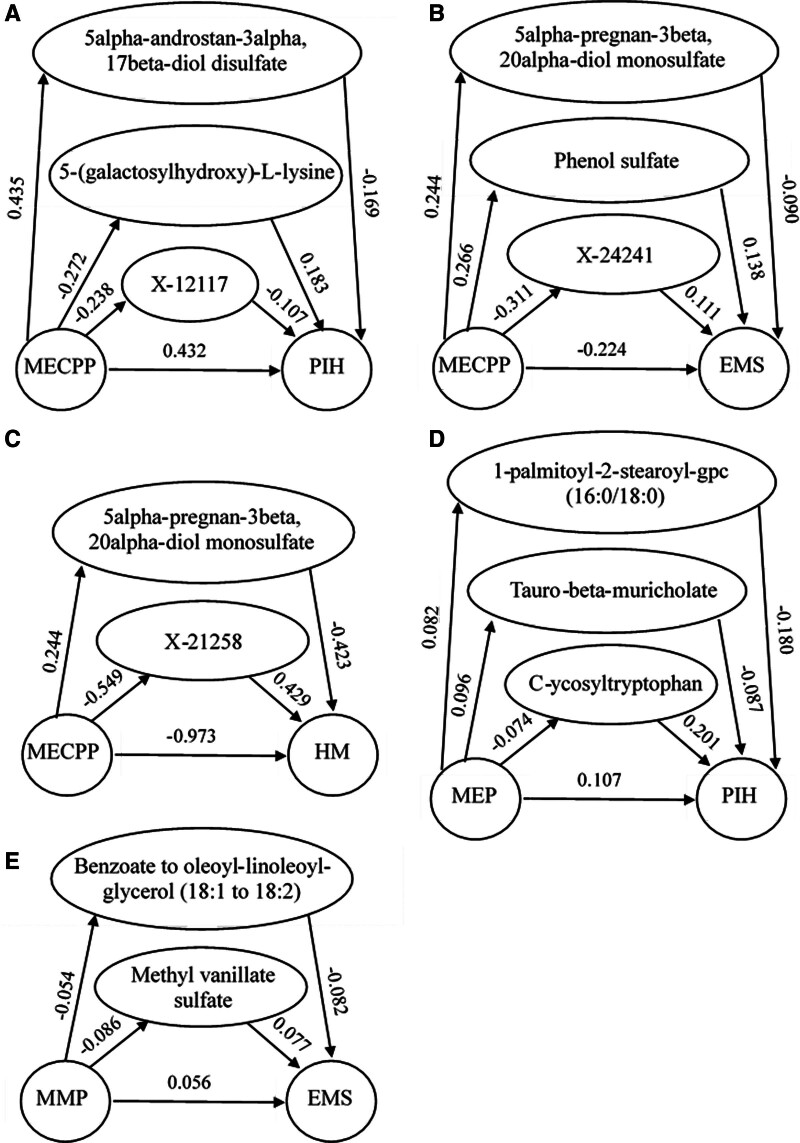
Mediating effect analysis in MR studies. Only the results with statistical significance were shown in the figure. The value on the arrow represents the beta coefficients (β). EMS = endometriosis, HM = hydatid mole, MECPP = mono-(2-ethyl-5-carboxypentyl) phthalate, MEP = mono-ethyl phthalate, MMP = mono-methyl phthalate, MR = Mendelian randomization, PIH = pregnancy-induced hypertension syndrome.

We found that the EDCs, which had statistically significant relationships with FRDs, were significantly associated with 178 blood metabolites. Specifically, MECPP, MEP, MMP, and BPA were associated with 47, 53, 51, and 27 blood metabolites, respectively. Further mediation analysis revealed that 13 blood metabolites modulated the relationship between EDCs and FRDs, as detailed in Figure 3.

The effect of MECPP on PIH was modulated by 5-(galactosylhydroxy)-l-lysine, 5α-androstan-3α,17β-diol disulfate, and X-12117 levels, with the corresponding mediating effects being −0.050, −0.074, and 0.026 respectively, and the corresponding direct effects were 0.482, 0.506, and 0.407, respectively. Then, the effect of MECPP on EMS was modulated by 5α-pregnan-3β,20α-diol monosulfate, X-24241 and Phenol sulfate levels, with the corresponding mediating effects being −0.022, −0.035, and 0.037, respectively, and the corresponding direct effects were −0.202, −0.189, and −0.260, respectively. Additionally, the effect of MECPP on HM was modulated by 5α-pregnan-3β, 20α-diol monosulfate, X-21258 levels, with the corresponding mediating effects being −0.103 and −0.236, respectively, and the corresponding direct effects were −0.870 and −0.737, respectively. Furthermore, the effect of MEP on PIH was modulated by Tauro-β-muricholate, C-glycosyltryptophan, 1-palmitoyl-2-stearoyl-gpc (16:0/18:0) levels, with the corresponding mediating effects being −0.008, −0.015, and −0.015, respectively, and the corresponding direct effects were 0.116, 0.122, and 0.122, respectively. Finally, the effect of MMP on EMS was modulated by Benzoate to oleoyl-linoleoyl-glycerol (18:1–18:2) ratio and methyl vanillate sulfate levels, with the corresponding mediating effects being 0.004 and −0.007, respectively, and the corresponding direct effects were 0.052 and 0.063, respectively.

## 4. Discussion

This extensive MR study investigated the causal relationships between 15 EDCs, 1400 metabolites present in plasma, and 11 FRDs. We found that certain EDCs (MECPP, MEP, MMP, and BPA) were associated with specific FRDs. Specifically, MECPP was associated with increased likelihood of PIH but with decreased likelihood of EMS and HM. MEP increased the risk of PIH, and MMP increased the risk of EMS. While the presence of the BPA lowered the likelihood of postpartum events. Moreover, 13 blood metabolites were identified to affect the relationship between EDCs and FRDs. The results exemplified the direct influence of EDCs on FRDs and highlighted the key mediation of systemic metabolic conditions while assessing the influence of EDCs on FRDs.

Many recent studies have explored the influence of MECPP and MMP on FRDs and obtained results similar to ours. For instance, a study conducted in Alberta found a significant link between exposure to MECPP and the incidence of PIH.^[9]^ A study involving case-control design in China indicated that there was a significant association between MMP levels in urine and EMS.^[10]^

Prior observational research regarding the connection between MEP and PIH also supported our results. An observational analysis in France, for instance, indicated that maternal exposure to certain phthalates, particularly MEP, significantly contributes to the onset of PIH.^[11]^ Furthermore, a study in the United States demonstrated that the exposure to harmful substances like MEP during the first to third trimesters of pregnancy is linked with variations in arterial pressure throughout the gestational period.^[36]^ Moreover, research carried out in Europe indicated an inverse relationship between systolic blood pressure and MEP levels, highlighting that as the concentration of MEP rose, the systolic pressure tended to decrease.^[37]^

As for our knowledge, there were no studies that assessed the links between MECPP and HM or BPA and PP. It is important to note that observed connections, including an increased likelihood of gestational diabetes and PCOS in women exposed to EDCs, were not found in this MR research.^[38-40]^ The differences could be because the data was from observational studies, the populations were diverse, or there was no causal relationship between the causes and outcomes. As a result, MR and similar approaches were needed to separate true effects from false ones.

ECDs can cause FRDs by disrupting hormones, creating oxidative stress and disturbing the body’s metabolism.^[8,41,42]^ For 1, exposure to phthalates may influence the renin-angiotensin-aldosterone system, reduce cortisol levels, affect how the thyroid system and hypothalamus-pituitary-adrenal axis function, alter DNA methylation, release inflammatory proteins, interfere with placental blood vessel growth, and injure vascular endothelium.^[42]^ Our findings corroborate these proposed mechanisms by identifying specific blood metabolites (e.g., X-12117 and 5-(galactosylhydroxy)-l-lysine) that mediate the association between EDCs and FRDs, thereby highlighting how metabolic pathways contribute to the reproductive toxicity caused by EDCs.

While prior research has established associations between EDCs, blood metabolic profiles, and the risks of hormone-sensitive conditions such as breast cancer,^[24]^ the mechanistic role of systemic metabolites in facilitating the effects of EDCs on FRDs requires further investigation. To the best of our knowledge, this research served as one of the earliest applications of MR to systematically identify specific blood metabolites as mechanistic intermediaries in the EDCs-FRDs relationship. This innovative approach advances our understanding of the molecular mechanisms underlying EDCs-related reproductive pathologies, offering novel insights into potential therapeutic or preventive targets.

These findings have significant public health implications. First, identifying EDCs as causal risk factors for FRDs underscores the need for stricter regulations on EDCs use in consumer products and industrial processes. Second, blood metabolites associated with EDCs exposure could serve as biomarkers for the early detection of reproductive risks. Third, understanding the mediating role of metabolites may inform interventions (e.g., dietary or pharmacological) to mitigate EDCs-induced reproductive harm.

By utilizing MR, this study effectively reduced confounding factors and reverse causality, thereby presenting robust findings that support cause–effect relationships. Meanwhile, integrating EDCs, metabolites, and FRDs offered a comprehensive understanding of the pathways linking environmental exposures to reproductive health. Notably, there were also several limitations. First, this study included primarily European populations, limiting its generalizability to other ethnic groups. Second, while IVW is a good primary estimator however it is not sufficient to establish a robust causal claim, and its validity is premised on the strong assumption that all instrumental variables are valid. Also, the supplementary methods do not prove causality but instead fundamentally test whether the causal inference remains stable when the stringent assumptions of the IVW method are relaxed. Third, the metabolome data may not capture all relevant pathways, and some metabolites may have been missed.

## 5. Conclusions

This research provides robust evidence supporting the cause–effect relationships between EDCs and FRDs, while also pinpointing specific blood metabolites as key mediators in these relationships. By integrating MR with metabolomics, we advance the understanding of the mechanisms underlying EDCs-induced reproductive harm and emphasize potential intervention points. These findings highlight the necessity of reducing EDCs exposure to protect female reproductive health and inform strategies for early detection and prevention of FRDs.

## Acknowledgments

Large scale data resources were made available by GWAS and related consortia. It is due to the financial backing of the National Natural Science Foundation of China.

## Author contributions

**Data curation:** Boyu Yin, Chunyu Li, Yi Pei.

**Formal analysis:** Boyu Yin, Chunyu Li.

**Funding acquisition:** Chunyu Li, Chuchao Zhu.

**Methodology:** Boyu Yin, Chunyu Li, Yi Pei, Chuchao Zhu.

**Project administration:** Huafang Wei, Lin Liang, Chuchao Zhu, Chengwen Song.

**Visualization:** Lin Liang.

**Writing – original draft:** Boyu Yin, Chunyu Li.

**Writing – review & editing:** Yi Pei, Huafang Wei, Lin Liang, Chuchao Zhu, Chengwen Song.

## Supplementary Material




