# Supplementary material for: Effects of endocrine disrupting chemicals and plasma metabolome on female reproductive diseases: A multidimensional Mendelian randomization study
Source: Medicine (Baltimore). 2026 Jan 16;105(3):e47248. doi: 10.1097/MD.0000000000047248 (PMC12826213; doi:10.1097/MD.0000000000047248)
Supplement: Supplementary file 1 [file medi-105-e47248-s001.docx]

**Supplemental material**

**Figure S1:**
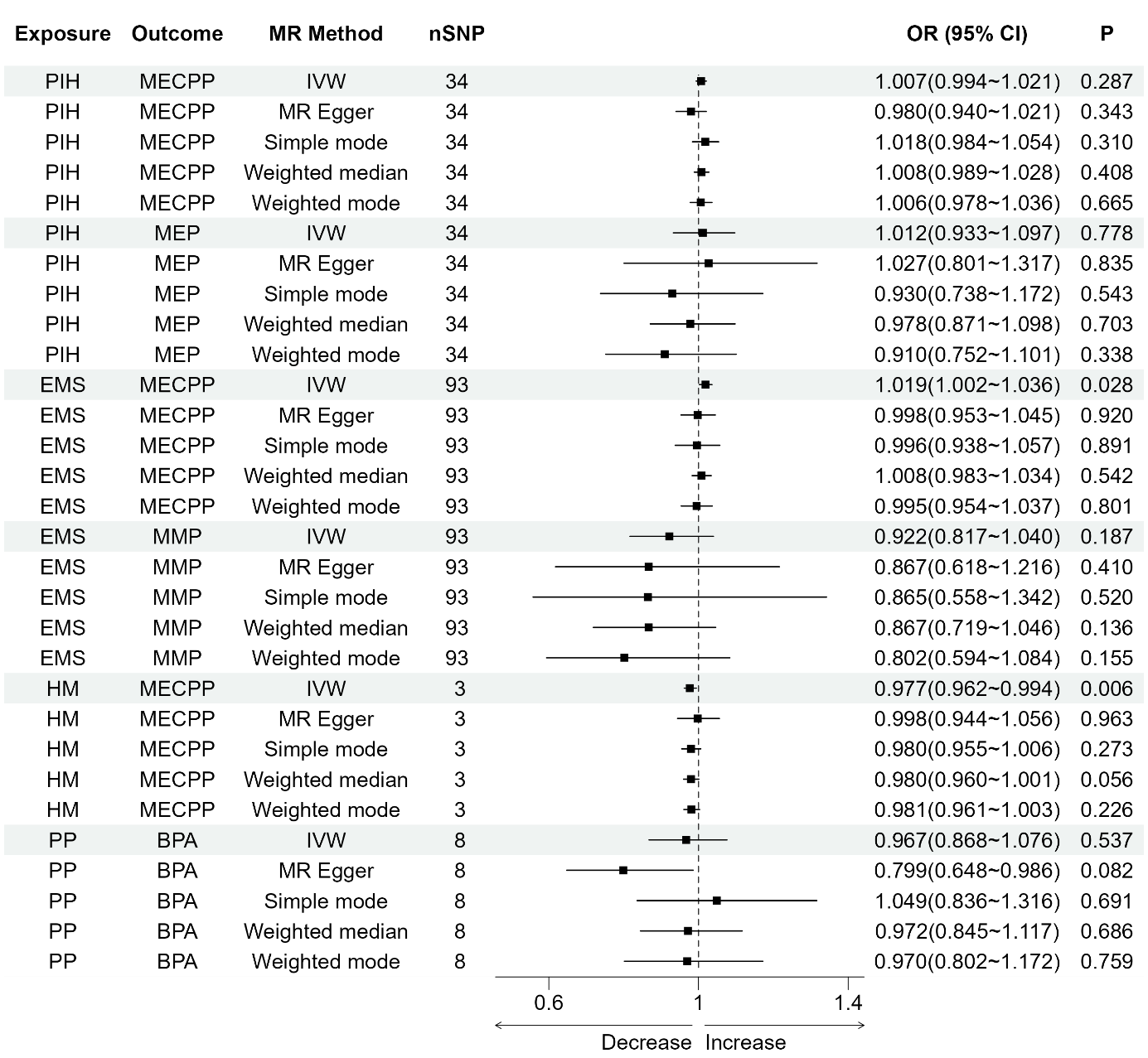


**Figure S1 MR Estimates of the effect of FRDs on EDCs.** MR: Mendelian Randomization; EDCs: Endocrine disrupting chemicals; FRDs: Female Reproductive Diseases; IVW: Inverse Variance Weighted; nSNP: number of Single Nucleotide Polymorphism; OR: Odds Ratio; CI: Confidence Interval. MECPP: Mono-(2-ethyl-5-carboxypentyl) phthalate; MEP: Mono-ethyl phthalate; MMP: Mono-methyl phthalate; BPA: Bisphenol A. PIH: Pregnancy-induced hypertension syndrome; EMS: Endometriosis; HM: Hydatid mole; PP: Placenta previa.

**Supplemental material**

**Figure S2:**
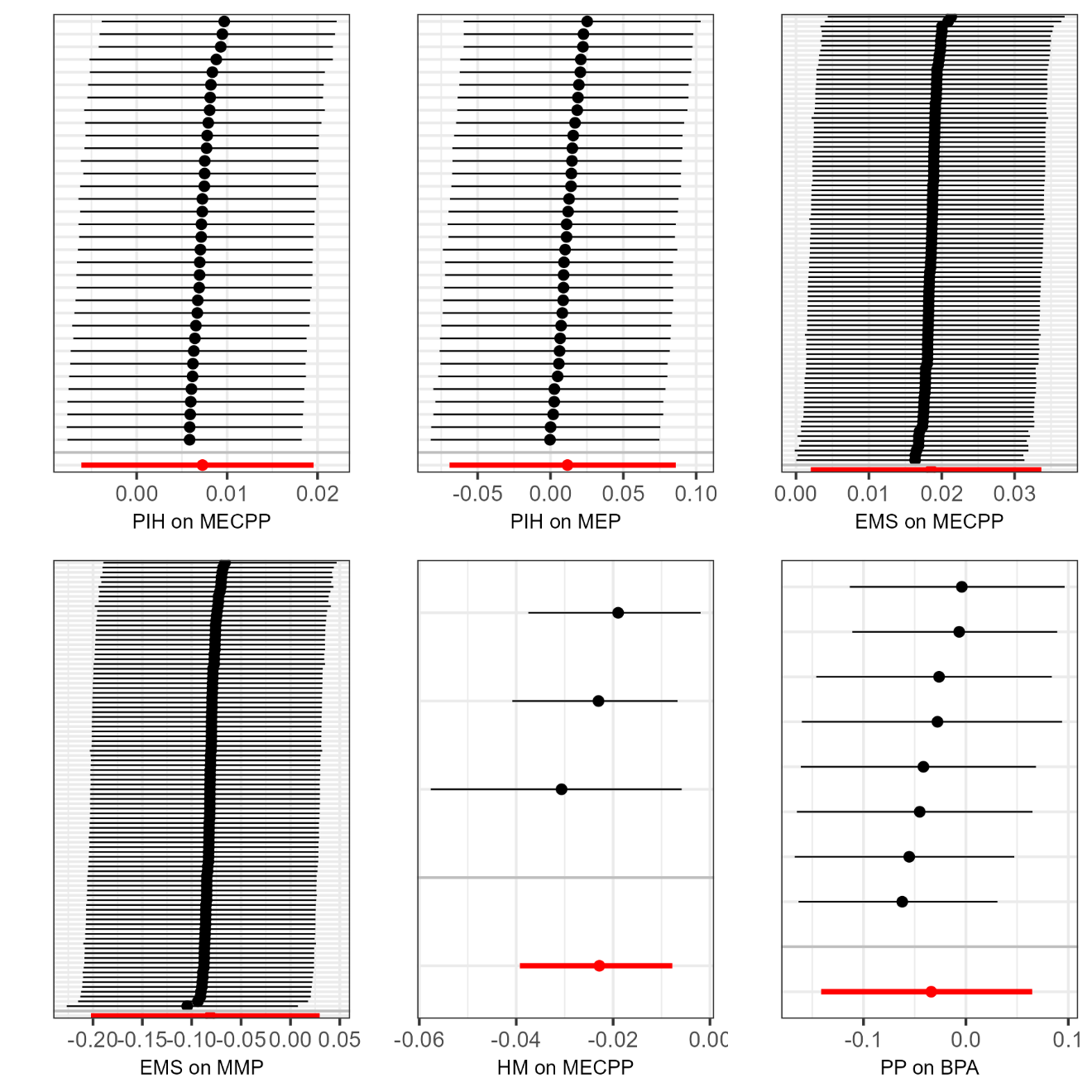


**Figure S2 Results of leave-one-out test of the effect of FRDs on EDCs.** EDCs: Endocrine disrupting chemicals; FRDs: Female Reproductive Diseases. MECPP: Mono-(2-ethyl-5-carboxypentyl) phthalate; MEP: Mono-ethyl phthalate; MMP: Mono-methyl phthalate; BPA: Bisphenol A. PIH: Pregnancy-induced hypertension syndrome; EMS: Endometriosis; HM: Hydatid mole; PP: Placenta previa.
