# Supplementary material for: Effects of endocrine disrupting chemicals and plasma metabolome on female reproductive diseases: A multidimensional Mendelian randomization study
Source: Medicine (Baltimore). 2026 Jan 16;105(3):e47248. doi: 10.1097/MD.0000000000047248 (PMC12826213; doi:10.1097/MD.0000000000047248)
Supplement: Supplementary file 2 [file medi-105-e47248-s002.docx]

**Supplemental material**

**Table S1:**

**Table S1 Heterogeneity and pleiotropy test of the MR Estimates.**

| **Exposure** | **Outcome** | **Heterogeneity** | |  | **Egger pleiotropy** | |  | **PRESSO global pleiotropy** | | |
| --- | --- | --- | --- | --- | --- | --- | --- | --- | --- | --- |
|  |  | **Q** | ***P*** |  | **Intercept** | ***P*** |  | **RSSobs** | ***P*** | **Outliers** |
| PIH | MECPP | 31.387 | 0.547 |  | 0.005 | 0.176 |  | 32.908 | 0.561 | None |
| PIH | MEP | 30.854 | 0.574 |  | -0.003 | 0.901 |  | 32.642 | 0.579 | None |
| EMS | MECPP | 96.909 | 0.343 |  | 0.002 | 0.344 |  | 105.434 | 0.277 | None |
| EMS | MMP | 70.610 | 0.952 |  | 0.005 | 0.703 |  | 78.896 | 0.914 | None |
| HM | MECPP | 0.792 | 0.673 |  | -0.014 | 0.579 |  | - | - | - |
| PP | BPA | 9.168 | 0.241 |  | 0.061 | 0.095 |  | 11.772 | 0.260 | None |

**Note:** Q represented Q statistic of heterogeneity test. RSSobs represented Residual Sum of Squares observed. **MECPP**: Mono-(2-ethyl-5-carboxypentyl) phthalate; **MEP**: Mono-ethyl phthalate; **MMP**: Mono-methyl phthalate; **BPA**: Bisphenol A. **PIH**: Pregnancy-induced hypertension syndrome; **EMS**: Endometriosis; **HM**: Hydatid mole; **PP**: Placenta previa.
